# Burst Stimulation for Sustained Locomotion Control and Autonomous Navigation of Terrestrial Cyborg Beetles

**DOI:** 10.34133/cbsystems.0537

**Published:** 2026-03-09

**Authors:** Hai Nhan Le, Huu Duoc Nguyen, Lachlan Fitzgerald, Peter Ross McAree, Hirotaka Sato, Tat Thang Vo-Doan

**Affiliations:** ^1^ School of Mechanical and Mining Engineering, The University of Queensland, Brisbane 4072, Australia.; ^2^ School of Mechanical and Aerospace Engineering, Nanyang Technological University, 639798, Singapore.

## Abstract

Terrestrial cyborg insects have become a leading candidate for insect-scale robots. These biohybrid systems utilize living insects as mobile platforms while controlling their locomotion via electrical stimulation of sensory organs. Despite remarkable advancement in locomotion control, the deployment of these cyborgs is hindered by their deterioration in locomotory responses to electrical stimulation, which typically involves prolonged, continuous stimuli. This study proposes burst stimulation as an alternative to the conventional stimulation protocol to achieve more sustained responses of cyborg insects from darkling beetles (*Zophobas morio*). Implementing burst stimulation enhances the sustainability of beetle’s responses, with the deterioration rate of the turning response to antenna stimulation reduced by 30%. The induced turning angle is 52% greater under burst stimulation, further advancing locomotion control. The graded response in turning is conserved when tuning the stimulation frequency from 10 to 40 Hz, promoting the integration of burst stimulation into feedback control systems for autonomous navigation of the cyborg beetle. The cyborg beetles are able to reliably and accurately navigate the defined path, with a 73% success rate and 12-mm tracking error. Furthermore, an in-depth analysis of navigation performance suggests that limiting the number of consecutive unilateral stimulations would enhance the efficiency and reliability of the navigation. This study demonstrates that implementing burst stimulation can effectively reduce habituation, improving the reliability, accuracy, efficiency, and sustainability of the control system for terrestrial cyborg insects, advancing the feasibility for real-world deployment.

## Introduction

Developing insect-scale terrestrial robots has long challenged roboticists and researchers. Achieving agile, dynamic, and robust locomotion in miniature robots [1] usually requires sophisticated mechanical designs and complex control algorithms [2,3], which are still outperformed by natural animals of similar sizes [4–6]. Furthermore, power constraints also limit the development and usability of these small-scale robots [7,8].

Biohybrid robots [5,9], specifically terrestrial cyborg insects [4], have emerged as a promising solution to address those challenges. Such biohybrid systems use living insects as robot platforms while controlling their movement through electrical stimulation of their neural or muscular systems by a miniature wireless backpack mounted on their body. The insects provide a lightweight but robust mobile platform with built-in sensors, actuators (muscles), legs, and an inner control system (central nervous system and brain) [10]. In addition, they possess agile locomotion and natural obstacle negotiation capabilities to adapt to various terrains dynamically [11–13]. These locomotory abilities facilitate the development of efficient navigation algorithms that focus on high-level control, leaving low-level control (sensory interfacing and actuation planning) to the insect’s nervous system [10]. Furthermore, locomotion control via electrical stimulation is energy-efficient, with only a few hundred μA current drawn to establish the movements of the insects [14–16], reserving the power for other artificial peripherals for various applications [6,17–20].

Over the past 2 decades, various insect species were used as robot platforms, such as darkling beetles (*Zophobas morio*) [15,21], flower chafers (*Mecynorrhina torquata*) [22,23], Madagascar hissing cockroaches (*Gromphadorhina portentosa*) [24–26], and migratory locusts (*Locusta migratoria*) [27,28]. Controllability was achieved over several locomotory actions in these insects, like turning [21,29,30], forward/backward movement [4,21], sideways motion [15], galloping/tripod gait walking [22], and jumping [27,28]. These controlled movements are attained through the electrical stimulation of the insects’ specific organs, either by eliciting locomotory responses by stimulating the ganglia [29] or by directly stimulating the leg muscles [14]. However, electrical stimulation of insect sensory systems, such as antennae, elytra, and cerci, has been a dominant method to induce desired motions of the cyborg insects due to its practicality and ease of implementation. The antennae play a crucial role in environmental navigation by perceiving directional cues such as air currents, tactile contact, and chemical signals, which lead to escape response [31–33] and obstacle avoidance [11,34]. When an antenna touches an obstacle, sensory receptors are activated and transmit information to the insect’s central nervous system [31,35,36], allowing it to determine the direction of the stimulus. This sensory input helps coordinate an asymmetric motor response, causing the insect to turn away from the obstacle. For example, if the left antenna detects a stronger stimulus, the insect may initiate a rightward turn [31]. Electrical stimuli are able to activate targeted organs by emulating natural cues via conductive electrodes implanted in the insects, creating a tissue–electrode interface with the sensory receptors [21,25]. This method has been implemented across terrestrial insects, such as darkling beetle (*Z. morio*) [21,37], Madagascar hissing cockroaches (*G. portentosa*) [25,38], and oriental migratory locust (*L. migratoria manilensis*) [28], utilizing input signals from their antennae for navigation and from elytra/cercus for triggering escape response.

Such high locomotion controllability enabled demonstrations of high-level navigation tasks of terrestrial cyborg insects. Not only open-loop point-to-point and path-following control [24,29] but also feedback-based control systems have been demonstrated with reliable and accurate autonomous navigation in cyborg insects [37,39]. By incorporating natural locomotory behaviors into navigation algorithms, the cyborgs successfully maneuvered through obstructed environments without relying on traditional obstacle-detection sensors [13]. Furthermore, integrating electronic sensors like IMUs (inertial measurement units) and ToF (time of flight) sensors helped predict and mitigate insects’ unexpected behaviors, enhancing navigation performance [39]. Other peripherals, such as RGB (standard digital red-green-blue) cameras, IR (infrared) sensors, and advanced technologies like neural networks and machine learning, have been incorporated to demonstrate the potential of terrestrial cyborg insects in practical applications such as navigating unknown environments, pipeline inspection, and human detection in search-and-rescue operations [6,39–41]. Exploration of swarm control in cyborg insects led to demonstrations of dispersion and leader–follower behaviors in irregular terrains filled with sand and debris [42].

Despite impressive technological advancements, the deployment of terrestrial cyborg insects remains limited by the unsustainability of their locomotory response to electrical stimulation. In specific, the induced motions tend to fade with repeated stimulation, leaving the insects uncontrollable. This phenomenon is often attributed to habituation, where the insects ignore repetitive signals, or to the impairment of tissue–electrode interfaces, reducing the impact of electrical stimuli [43–45]. Various approaches have been proposed to address this challenge, including monitoring electrode–tissue interface impairment [46], implanting electrodes at the early morphological stage to enhance the interface [47], or optimal stimulation parameters to mitigate habituation [48]. A more sophisticated approach involved adjusting stimulation voltage based on the magnitude of the insect’s behavioral response and restoring normal behavior after habituation using high-voltage stimulation [38].

Since sensory systems often fire in bursts [31,49], modulating electrical stimuli in burst form may more closely emulate the sensory signal of the antenna, inducing turning in insects more effectively. Modulating the stimulation signal has been shown as a potential method to enhance the sustainability of locomotion control in cyborg insects, particularly transforming a prolonged stimulus (continuous stimulation) into a burst of short stimuli (burst stimulation) [50]. Existing locomotion control protocols for cyborg insects typically employ square pulse trains for stimulation [25,28,48], which prolonged stimuli tends to degrade the insects’ response over time, while short stimuli often fail to elicit meaningful reactions [25]. Intuitively, a burst stimulus, comprising short stimuli interspersed with nonstimulation intervals, may offer a solution. This stimulation waveform was reported to produce more natural and gentle reactions in cyborg fish [51] and improve flight control in cyborg beetles (*M. torquata*) [52]. However, in-depth research in the field of terrestrial cyborg insects remains lacking. In spite of preliminary discussion for darkling beetles (*Z. morio*) [50] and Madagascar hissing cockroaches (*G. portentosa*) [38], the effect of burst stimulation on autonomous locomotion and navigation control of terrestrial cyborg insects requires further investigation.

**Fig. 1.**
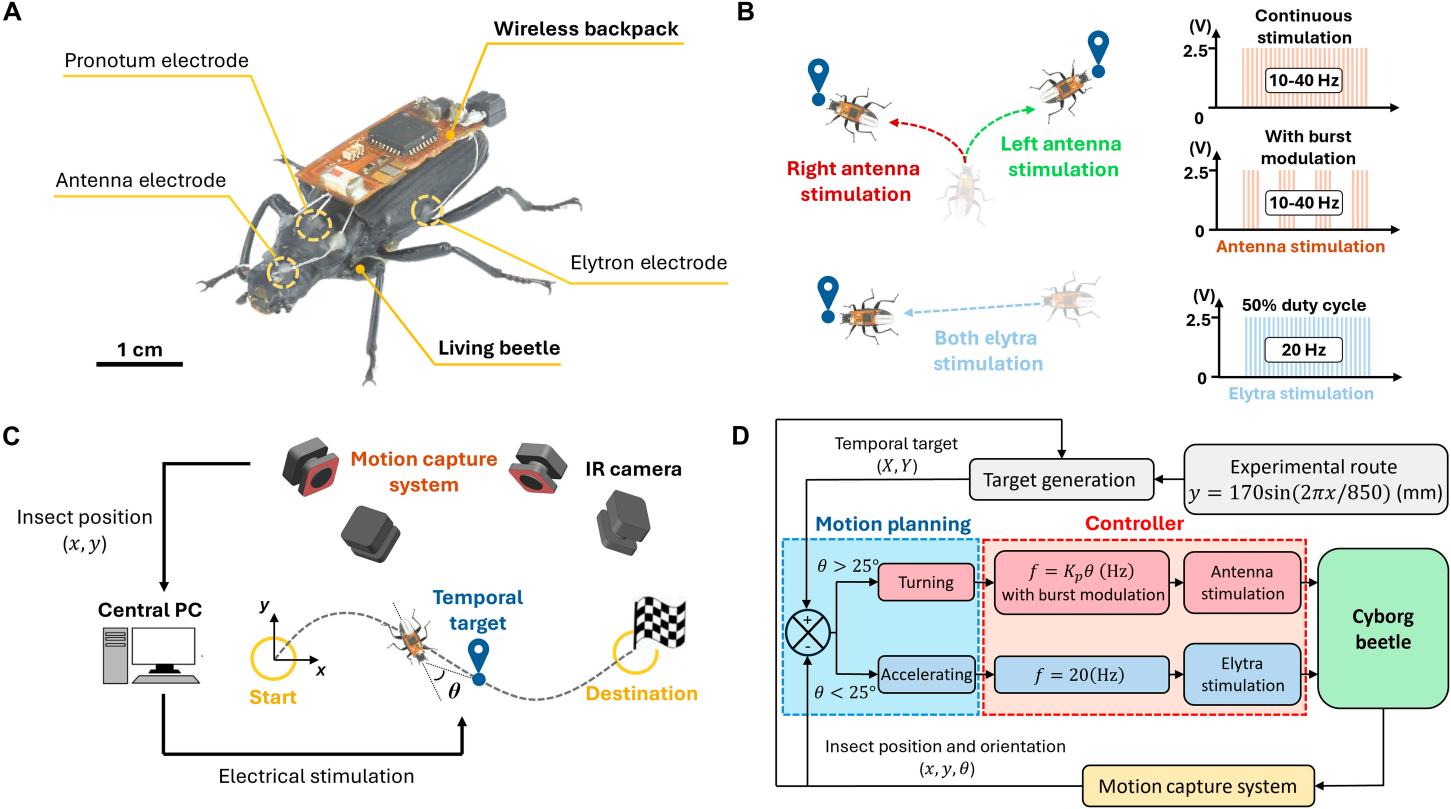
Experiment setup for autonomous navigation of terrestrial cyborg beetles with burst stimulation. (A) The cyborg is made of the darkling beetle *Z. morio* with electrodes implanted into its antennae and elytra and a wireless backpack mounted on its back. (B) Stimulation protocols for locomotion control. Electrical stimulation of the left antenna induces a right turn and vice versa, while stimulating both elytra drives the beetle forward. The upper right panel illustrates the traditional continuous protocol, and the middle panel represents the proposed burst modulation. The lower part shows the electrical waveform for elytra stimulation. (C) Overview of the feedback control system for autonomous navigation of cyborg beetles. The beetle’s motion was limited within an experimental region of 1,200 mm × 600 mm when being navigated to track a predetermined path of *y* = 170sin(2𝜋𝑥/850) mm. In each navigation trial, the beetle is initially placed at the starting region (𝑥 = 0 mm, *𝑦* = 0 mm, *R* = 40 mm) and navigated toward the destination region while tracking the predetermined path (𝑥 = 0 mm, *𝑦* = 850 mm, *R* = 40 mm). The beetle’s instantaneous location data (𝑥,*y*) are tracked by a motion capture system (Vicon Bonita). (D) Block diagram of the feedback control system.

Herein, this study investigates the impact of burst stimulation on the sustained controllability of terrestrial cyborg insects, specifically those using darkling beetles (*Z. morio*) as mobile platforms (Fig. 1A). By analyzing the beetle’s responses to continuous stimulation, the study hypothesizes that burst stimulation may improve autonomous controllability for long-term operation. A comparison between 2 methods reveals that burst stimulation enhances the sustainability of the beetle’s response to the antenna stimulation, reducing the decay rate of the induced turning angle by approximately 30%. Furthermore, burst stimulation elicits a more profound response of approximately 52% than continuous stimulation while delivering the same electrical charge, further prolonging the response sustainability and highlighting the efficiency of this protocol. In addition, burst stimulation elicits a graded response to stimulation frequency, enabling its use for autonomous navigation of terrestrial cyborg insects. Finally, a close look at navigation performance reveals potential strategies for improving reliability and success rates, such as limiting repetitive unilateral stimulations. As a result, this study contributes a novel solution to achieving sustained control in terrestrial cyborg insects, advancing the development of these biohybrid systems for applications beyond the laboratory.

## Materials and Methods

### Terrestrial cyborg insects

Small and lightweight darkling beetles (*Z. morio*) (approximately 0.5 to 0.9 g, 2.5 to 3 cm) were selected for this study (Fig. 1A). Such a beetle has demonstrated its high locomotion and navigation controllability [53]. Beetle colonies were reared in a compartmentalized housing system (approximately 9,000 cm^3^ per compartment, NexGen Mouse 500, Allentown) and fed with water, carrots, and other vegetables weekly. The housing system maintained a temperature of 25 °C and 60% relative humidity. Given the species’ lifespan of approximately 3 months, young beetles (1 to 2 months old) were selected for experiments to minimize the impact of aging [54].

While standard ethical regulations for invertebrate and biohybrid research are still under development [55,56], this study ensured wellbeing and appropriate treatment for the beetles throughout the experiments. Environmental conditions, including temperature, humidity, and nutrition, followed established guidelines [57]. The housing system was cleaned weekly to maintain hygiene. Each colony housed approximately 20 beetles to ensure adequate space. Post-experimental beetles were detached from implanted electrodes and treated as intact ones. The beetles were anesthetized before electrode implantation and removal, regardless of the ongoing debate about their capacity to feel pain [58].

The electrode implantation procedure followed established methods [15,21,37]. Four working electrodes and one common electrode were implanted to control the beetle’s turning and acceleration (Fig. 1A and B). Prior to implantation, the beetle was anesthetized with CO_2_. The common electrode was implanted in the pronotum. Two working electrodes were inserted into the antennae to control turning, and the remaining 2 were inserted into the leading edges of the elytra to elicit forward acceleration. All electrodes were secured with beeswax, and the implanted beetle was allowed 2 to 4 h rest before experimentation.

The terrestrial cyborg beetle was equipped with an electronic wireless backpack attached to the beetle’s back using double-sided tape (Fig. 1A and Fig. S1) [37]. This backpack, designed with a Texas Instruments CC2650 microcontroller, supports Bluetooth communication (up to approximately 10-m range) and features 8 stimulation channels with adjustable parameters (e.g., frequency). The backpack was powered by a rechargeable 3.7-V, 8-mAh lithium-ion battery.

### Pulse train for continuous and burst stimulation

The elytra stimulation was conducted using a conventional fixed rectangular stimulus (2.5-V amplitude, 20-Hz frequency, 200-ms duration, 50% duty cycle) based on parameters from previous studies that elicited significant locomotory responses in the beetle’s forward acceleration (Fig. 1B) [15,37]. The antenna stimulation employed a similar continuous pulse train, named “continuous stimulation”, with a 2.5-V amplitude, 2-ms pulse width, and 400-ms duration (Fig. 1B). The stimulation frequencies ranged from 10 to 40 Hz, eliciting a graded turning response in the beetle [37,50]. The lower bound of 10 Hz was the lowest frequency ensuring a significant turning response, while the frequency above the upper bound of 40 Hz was reported to result in a downtrend of turning angle [21].

In addition to this conventional stimulation protocol, a new “burst stimulation” protocol was developed, comprising four 100-ms bursts separated by 50-ms stimulation-free periods. Both protocols used the same stimulation parameters: 2.5-V amplitude, 2-ms pulse width, and 10- to 40-Hz frequency (tuned by the P controller of the feedback control system). Because the time-averaged power consumption is identical for the 2 protocols, burst stimulation does not reduce overall battery life. From the signal modulation perspective, the burst stimulation signal was generated by modulating the continuous signal with a ~6.7-Hz carrier signal (66% duty cycle, 100-ms on-time). Despite their structural differences, both protocols delivered the same total stimulation intensity (400 ms of active charging) to the beetle, ensuring that the insect responses under both the traditional continuous waveform and the burst stimulation in this study are comparable. This allows for accurate conclusions about which stimulation method is more effective.

### Feedback control system for autonomous navigation

The feedback control system was adapted from previous studies, which established autonomous path-following navigation in terrestrial cyborg insects [37]. Using a carrot-chasing approach, this system enabled the beetle to maneuver autonomously along arbitrary, predetermined paths. Multiple temporal targets were generated along the path, and as the beetle approached each target, a new one was established. This pursuit continued until the beetle reached its final destination. To navigate the beetle toward each target, the control system periodically monitored its orientation error, i.e., the acute angle *θ* between the beetle’s orientation and its temporal target, to adjust the stimulation signal accordingly (Fig. 1C and D). The monitoring period was defined as the update interval. The system employed 2 controllers, activated based on the orientation error. When the beetle faced the target (*θ* ≤ 25°), a thrust controller accelerated the beetle by stimulating its elytra. Otherwise, a proportional controller, i.e., P controller, steered the beetle using the antenna stimulation. The stimulated antenna was selected to steer the beetle toward the target along the acute angle *θ*. The P controller modulated the stimulation frequency proportionally to the orientation error: *f* = *K*_p_ · *θ*, where *f* is the stimulation frequency and *K*_p_ is the proportional gain (Fig. 1D). The output signal was then modulated with a ~6.7-Hz carrier signal to generate the burst stimulation.

### Experiment setup

Experiments were conducted to collect the beetle’s locomotory behavior under burst stimulation and evaluate its feasibility for autonomous navigation. The experimental setup was inherited from prior research on terrestrial cyborg insects [37]. The control system was used to guide the beetle along a predetermined sine curve path, described by the equation *y* = 170sin(2π*x*/850) (mm), from origin to destination, each marked by a 40-mm radius circle (Fig. 1C). Each navigation trial was terminated when the beetle reached the destination, exited the experimental region (120 cm × 60 cm), or after 5 min, whichever occurred first. Trials were classified as successful or failed based on whether the beetle reached the destination before termination. To avoid bias, the origin and destination were swapped between consecutive trials. Each beetle underwent 12 trials with 5-min rest periods between trials.

Experimental trials were conducted under loosely tethered conditions to extend the experiment duration [37]. A 1.5-m-long copper wire (44 AWG, Remington Industries) was used to transmit electrical signals from the stimulator to the beetle. The feedback control program was written in MATLAB and embedded in a main PC connected to the stimulator. A 3-dimensional (3D) motion capture system (Vicon) operating at 100 fps monitored the beetle’s motion, providing real-time feedback to the control program and logging data for post-experiment analysis (Fig. 1C). The control system was evaluated using combinations of 2 control parameters: update interval, set at 1.0 and 1.5 s, and proportional gain *K*_p_ set at 0.5.

### Data analysis

**Fig. 2.**
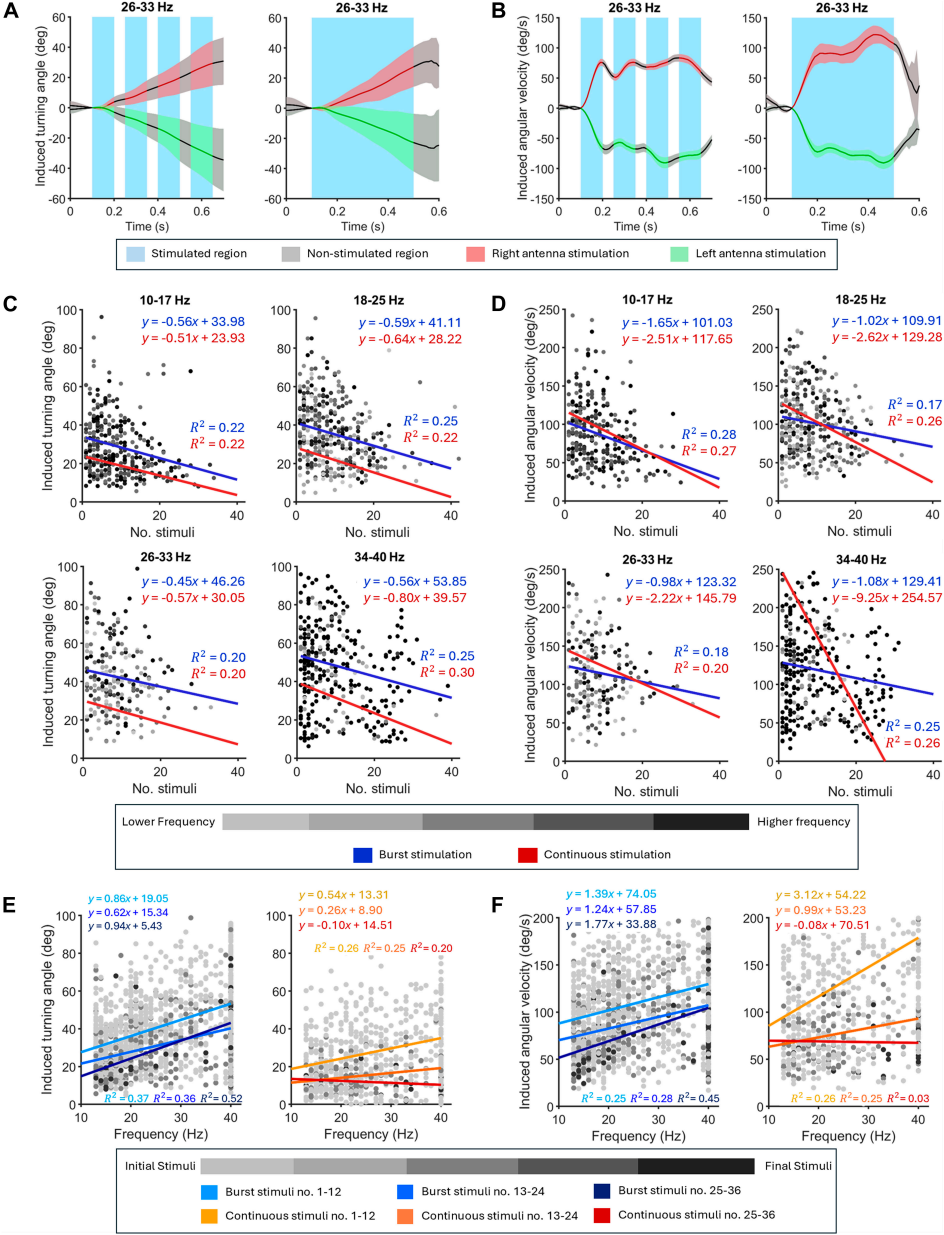
Sustained locomotory responses of terrestrial cyborg beetles with burst stimulation. (A and B) Representative profiles of the induced turning angle and angular speed during burst stimulation (left) and continuous stimulation (right) in the frequency range of 26 to 33 Hz. Data are shown as mean ± SE, i.e., colored lines and shaded regions. The peak of angular speed occurred after 100 ms in a 400-ms-long continuous stimulation inspires burst stimulation with 4 short 100-ms bursts. (C and D) Reduction of induced turning angle and angular velocity when increasing the stimulation number in 4 frequency groups (*N* = 5 beetles, *n* = 1,272 stimulations). Blue and red lines indicate the linear regression of the induced response versus the stimulation order. Statistically, burst stimulation slows down the reduction rate (i.e., enhanced response sustainability) and generally provides greater induced turning angles compared to that of continuous stimulation (i.e., efficient locomotion control). (E and F) Graded responses in induced turning angle/angular speed and their diminishing over extended stimulation. The data are sorted into 3 groups based on the stimulation order, including 1st to 12th, 12th to 24th, and 25th to 36th stimuli. Blue and red lines indicate linear regression of the induced response versus stimulation frequency. Light colors indicate stimulation in groups of early stimulation, while dark ones indicate later stimulation. Like continuous stimulation, burst stimulation induces graded responses to the stimulation frequency. Beyond continuous stimulation, burst stimulation maintains such relationships even in late stimulations.

The beetle’s locomotory responses to burst stimulation of its antennae and elytra were extracted from the navigation results and smoothed using a mean filter with a 0.1 sliding window (i.e., at each instant, the filtered value represents the mean of all samples acquired during the centered 0.1-s interval). By averaging over this short time span, rapid frame-to-frame noise is attenuated, while slower, behaviorally meaningful trends in the signal are preserved. Linear velocity, representing the induced forward acceleration, was calculated from the beetle’s response to its elytra stimulation. The beetle’s turning response to its antenna stimulation under continuous stimulation was evaluated based on previous data [37]. The induced turning angle and angular velocity were calculated to quantify the impact of the antenna stimulation (Fig. 2A and B and Figs. S2 and S3). The induced value is the difference in heading angle or angular velocity compared to that right before the stimulation. The heading angle is calculated based on the vector connecting the head–tail coordinates of cyborg beetles. For burst stimulation, the induced angular velocity was calculated as the mean of each burst’s peak angular velocities (Fig. 2B and Fig. S2). For continuous stimulation, it was the final angular velocity at the end of the stimulation period (Fig. 2B and Fig. S3). Left and right turns were denoted by negative and positive values, respectively. In the analysis of the graded response, the beetle’s responses to the antenna stimulation were categorized into 4 groups according to the stimulation frequencies used, including 10 to 17 Hz, 18 to 25 Hz, 26 to 33 Hz, and 34 to 40 Hz. For the sustained response analysis, the responses were categorized into 3 groups based on stimulation order, including 1st to 12th, 13th to 24th, and 25th to 36th stimuli. Responses outside the range of ±2.7*σ* (where *σ* is the standard deviation of each group) were excluded as outliers. Student’s *t* test (*α* = 0.05) was used to compare the beetle’s responses between the 2 stimulation protocols. Spearman’s rank correlation and linear regression were used to evaluate the graded and sustained responses. Although some of the linear regression correlations are relatively weak, they offer a straightforward and consistent way to visually compare the effects of burst and continuous stimulation. We acknowledge that linear regression is not ideally suited to model the nonlinear deterioration of turning responses or the complex frequency–response relationships inherent to living insects. However, the purpose of this analysis is not to develop a predictive model, but rather to provide a comparable visualization of response trends across the 2 stimulation protocols.

Navigation performance was evaluated using 4 metrics: success rate, tracking error, navigation time, and navigation effort [37]. Success rate, representing the control system’s reliability, was calculated as the percentage of successful trials out of the total conducted. Tracking error, indicating the path-following accuracy, was estimated by dividing the area between the actual trajectory and the predetermined path by the path length [59]. Navigation time and effort, used to assess control system efficiency, were calculated as the average time and number of stimuli for successful trials. The navigation experiment included *N* = 5 beetles with *n* = 60 trials. Four trials (6.7%) were excluded from the analysis due to significant positional data loss (more than 5 stimuli with missing tracking data). Seven trials were terminated prematurely due to human error and were classified as failed navigations in the analysis, i.e., near-complete trials. Specifically, these autonomous navigation trials were unintentionally terminated due to an interruption during control system shutdown, after which the beetles received no further control commands to guide them toward the destination. Although these trials were initially considered for exclusion from the primary analysis, they were ultimately retained because similar random loss-of-control events may realistically occur in real-world deployments. Moreover, in all 7 trials, the beetles successfully tracked at least three-quarters of the predefined path before control was lost. These near-complete trials can therefore be regarded as intermediate cases between fully successful trials and “complete-deviation” failures. Following the loss of control, the cyborg beetles could either eventually reach the destination or escape the navigation arena under continued unilateral antennal stimulation.

## Results and Discussion

### Sustained and graded locomotory response induced by burst stimulation

Controlled locomotion of terrestrial cyborg insects under continuous stimulation often deteriorated over time. In darkling beetles (*Z. morio*), the magnitude of the turning response to antenna stimulation decreased with repeated stimuli (Fig. 2C and D). Specifically, at the stimulation frequency of 10 to 17 Hz, the induced turning angle exhibited a reduction rate of −0.51°/stimulus, which became faster with higher frequencies, reaching up to −0.80°/stimulus at 34 to 40 Hz (linear regression, *R*^2^ > 0.2, Spearman’s correlation, *N* = 20 beetles, *n* = 236 stimuli, *P* < 0.05, *ρ* < −0.15; Fig. 2C). The maximum induced angular velocity followed a similar trend, with its average value decreasing by approximately 80% and sharply dropping by 100% as the number of stimuli doubles within the frequency ranges of 18 to 25 Hz and 34 to 40 Hz, respectively (linear regression, *R*^2^ > 0.17, Spearman’s correlation, *N* = 20 beetles, *n* = 236 stimuli, *P* < 0.001, *ρ* < −0.21; Fig. 2D). The graded response of the beetle was also affected. While the induced turning motion could typically be graded, with turning angle and angular velocity increasing in tandem with stimulation frequency, this relationship was disrupted with repeated stimulation (Fig. 2E). For example, within the first 12 stimuli, the induced angle increased by 17° as the frequency rose from 10 to 40 Hz. However, this change diminished to 9° between the 25th and 36th stimuli (linear regression, *R*^2^ > 0.2, Spearman’s correlation, *N* = 20 beetles, *n* = 48 stimuli, *P* < 0.05, *ρ* > 0.24; Fig. 2E). Similarly, the slope representing the relationship between stimulation frequency and maximum induced angular velocity decreased from 3.12 to −0.08 (approaching horizontal) as the number of stimuli increased from 12 to 36 (linear regression, *R*^2^ > 0.03, Spearman’s correlation, *N* = 20 beetles, *n* > 48 stimuli, *P* < 0.05, *ρ* < 0.2; Fig. 2F). For the continuous stimulation condition in Fig. 2E and F, the low *R*^2^ values highlight a key limitation of the continuous protocol. Specifically, after repeated stimulation, the graded relationship between input frequency and output turning response diminishes, leading to weaker correlations.

The deterioration in the beetle’s response negatively impacted its controllability, reliability, and accuracy in autonomous navigation. As the beetle’s response diminished, the control system lost control over its motion. A feedback-based navigation system would attempt to counter the reduced response by increasing the control signal, e.g., the stimulation frequency [37]. However, this action became less effective because the reduction rate tends to be greater at higher frequencies (Fig. 2C and D). Furthermore, because the feedback-based navigation relied on the graded response of the beetle [14,15,21], its performance (success rate and accuracy) would be compromised when this graded feature diminished over time. Replacing conventional continuous stimulation, a 400-ms-long pulse wave, with burst stimulation would potentially sustain the beetle responses by providing recovery durations (Fig. 2A and B). The burst stimulation strategy consisted of 4 short 100-ms stimuli, separated by 50-ms stimulation-free periods. These intervals were chosen based on observations that the angular velocity induced by continuous stimulation peaks after approximately 100 ms before declining by about 10% [50]. This 600-ms burst stimulation delivered the same amount of electrical charge (stimulation intensity) to the beetle’s antenna as its 400-ms continuous counterpart. From a signal processing perspective, the burst stimulation was generated by modulating the continuous stimulation with a carrier signal of approximately 6.7-Hz frequency and a 66% duty cycle.

The burst stimulation strategy enhanced the sustainability of locomotion control in terrestrial cyborg insects by mitigating the decline in induced turning response that came about from repeated stimulations (Fig. 2B). At a stimulation frequency of 18 to 25 Hz, replacing continuous stimulation with burst stimulation reduced the decline in induced turning angle by 7.8%, from −0.64°/stimulus to −0.59°/stimulus (linear regression, *R*^2^ > 0.22, Spearman’s correlation, *N* = 5 beetles, *n* = 383 stimuli, *P* < 0.001, *ρ* < −0.21; Fig. 2C). This sustained response with burst stimulation became even more pronounced at higher frequencies. At 34 to 40 Hz, the decline in induced turning angle and maximum angular velocity were slowed by 30% and 88.3%, respectively (Fig. 2C and D). In addition, the induced turning angle under burst stimulation was greater than that under continuous one, further enhancing the sustainability of the beetle’s response (Fig. 2E). Specifically, the average induced turning angle increased by approximately 52% with burst stimulation, e.g., it rose from 23.1° to 35.5° at 18 to 25 Hz (*t* test, *n* > 200 stimuli, *P* < 0.001). Importantly, burst stimulation also preserved graded response in the beetle (Fig. 2E and F), making it suitable for developing feedback-based control system in cyborg insects. Particularly, the induced turning angle and maximum angular velocity could be graded from 28° to 53° and from 88°/s to 130°/s, respectively, by adjusting the stimulation frequency from 10 Hz to 40 Hz (linear regression, *R*^2^ > 0.25, Spearman’s correlation, *N* = 5 beetles, *n* = 921 stimuli, *P* < 0.001, *ρ* = 0.37; Fig. 2E and F). This relationship, observed in the first 12 stimuli, persisted as the number of stimuli increased, overcoming the limitation of continuous stimulation. Even at the 25th stimulus, the induced angular velocity and turning angle were still graded by 190% and 103%, respectively, by altering the stimulation frequency from 10 to 40 Hz. Although continuous stimulation results in the beetle adjusting heading angle rapidly with high angular velocity, there are potential risks of tissue damage [24] and habituation [44], which might lead to low turning response in prolong use. The short rest periods in burst stimulation allow the insect to maintain movement in the desired direction at approximately the same speed with the same amount of electrical charge transferred to its antenna. This results in higher turning angles as compared to continuous stimulation (Fig. 2C) while minimizing the risk of tissue damage, despite the latter exhibiting higher angular velocities (Fig. 2D).

Such enhancement in the sustainability of locomotion control demonstrated that burst stimulation was an effective alternative to the conventional continuous one in terrestrial cyborg insects. This strategy not only slowed the deterioration of the beetle’s response but also established a higher initial response level than continuous stimulation, thus prolonging the insect’s controllability. Furthermore, burst stimulation provided a graded response that persisted with repeated stimuli, enabling the development of more efficient feedback-based control systems that maintained performance over extended operation times. The advantages of burst stimulation may be attributed to its resemblance to natural neuronal firing patterns, where electrical pulses are delivered in short, repetitive bursts rather than continuously [51,52]. By aligning more closely with these endogenous firing patterns, burst stimulation enhances synaptic transmission [49], potentially supports more efficient motor neuron recruitment, and reduces fatigue through partial recovery during interburst intervals. Additionally, burst stimulation may reduce the accumulation of electrical charge and improve charge dissipation during stimulation-free periods, potentially minimizing tissue–electrode damage [51], although further investigation is needed to confirm this. As this stimulation complements existing research on sustaining locomotory responses in cyborg insects, it could be integrated with other approaches. For instance, it could replace the continuous signal used in the stimulation schemes that alternate voltage to reduce habituation in cyborg cockroaches [38]. Modulating rectangular pulse waves into analog-like signals can increase the success rate of inducing turning motions in cyborg cockroaches [48]; applying an additional modulation layer to create burst stimulation may further enhance the success and sustainability of the stimulation protocol. Further optimization of burst stimulation parameters, such as the stimulation-free period, may extend the beetle’s response even more.

### Autonomous navigation of cyborg beetles using burst stimulation

**Fig. 3.**
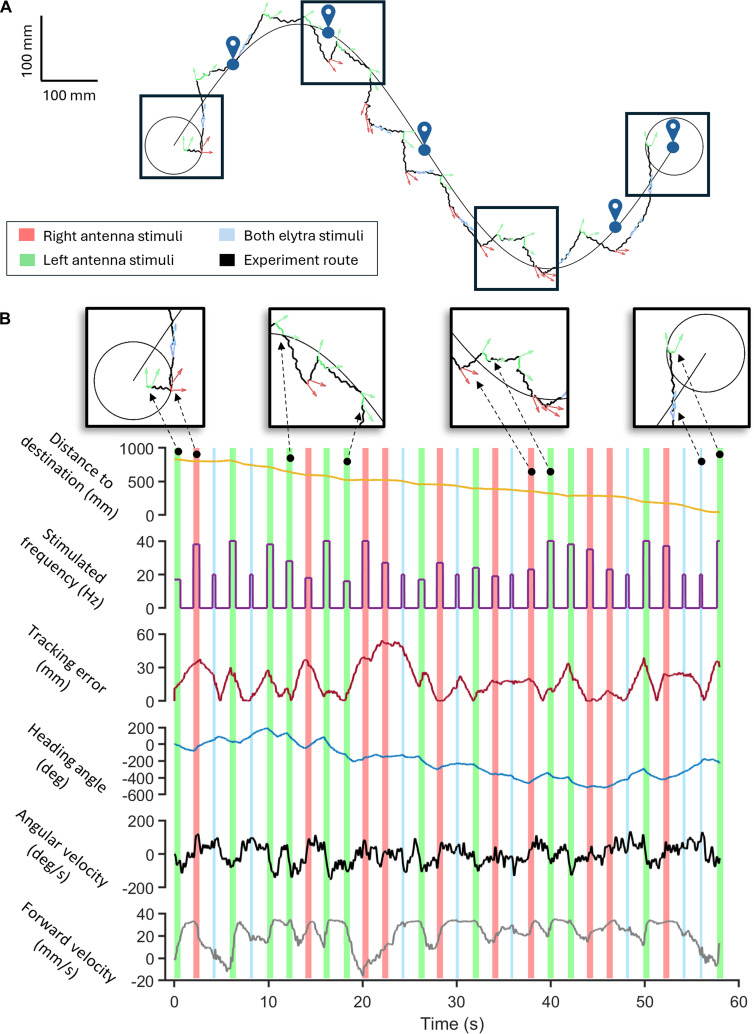
Representative demonstration of successful path-following control in terrestrial cyborg beetles. (A) The beetle’s trajectory is displayed together with electrical stimulation issued by the feedback control system, with red, green, and blue representing stimulations of the right and left antennae and both elytra, respectively. Arrows indicate the heading of the beetle. (B) A closer look at the navigation performance. The top row shows zoom-in windows of the beetle’s response along the path. The second row (dark yellow line) shows the instantaneous distance between the beetle and the center of the destination region. The third row (purple line) presents stimulation frequencies and stimulation sites. The fourth row (dark red line) shows the instantaneous tracking error between the beetle trajectory and the experimental route. The fifth row (blue line) presents the instantaneous heading angle. The fifth and sixth rows (black and gray lines) are instantaneous angular and forward velocities. Generally, in successful navigation, the control system alters the stimulated position based on the relative position of the beetle to the predetermined sine curve. Stimulation frequency is changed to correct the beetle orientation, reflected by the follow-up reduction in the tracking errors.

**Fig. 4.**
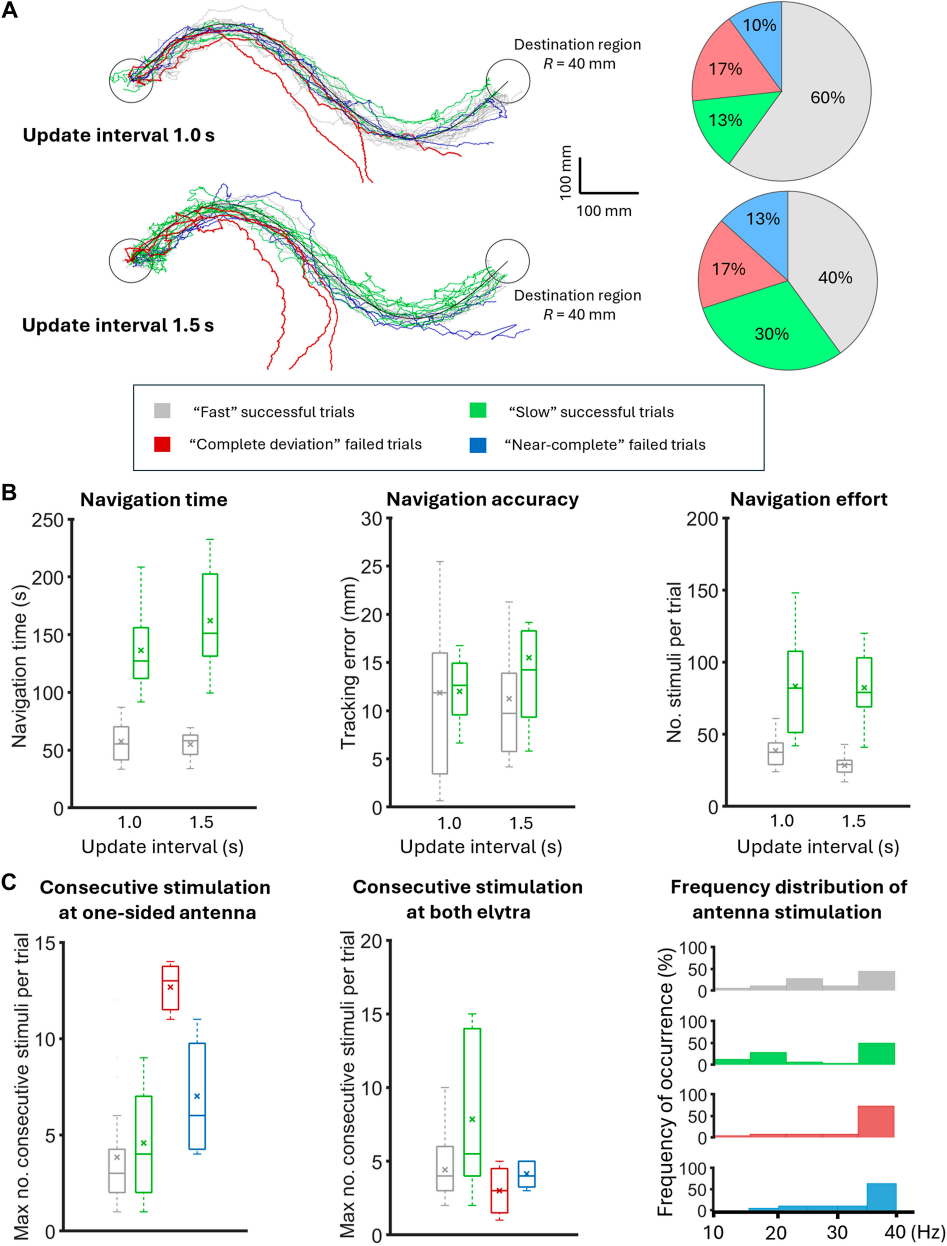
Performance of the feedback control system with burst stimulation for autonomous navigation of terrestrial cyborg beetles. (A) Overview of successful and failed navigation (*N* = 5 beetles, *n* = 56 trials). The overlay of all trajectories (left) and the ratio of successful and failed navigations (right) under 2 update intervals (1.0 and 1.5 s) are displayed. As introduced, the starting point of the beetles is alternated between the left and right ends of the path to avoid navigation bias. Herein, those starting from the right are flipped 180° for consistency in presentation. “Fast” successful trials (gray) are defined as those that complete the path within 90 s, while “slow” successful ones (green) complete the path in more than 90 s. “Near-complete” failed trials (blue) are when the beetle partially tracks the path but fails to reach the final destination, and “Complete deviation” failed trials (red) are when the beetle significantly deviates from the path. Generally, the feedback system can attain reliable navigation with a more than 70% success rate. “Fast” successful trials can be increased with a short update interval of 1.0 s. (B) Evaluation of successful navigation with different metrics. Data are displayed as boxplots, with the cross marks representing means and horizontal lines representing medians. Both “fast” and “slow” successful trials result in similar tracking accuracy, but the former is favored for its timely and efficient aspects. (C) A closer look at consecutive stimulation. This phenomenon appears unilaterally in the antenna stimulation (left panel), appearing to cause “complete deviation” in failed navigations, i.e., they occur frequently in these trials. All successful and failed navigations share similar consecutive stimulations in elytra stimulation (middle panel). The distribution of stimulation frequencies (right panel) spread across its spectrum in successful navigations with a slight skew toward high frequencies, suggesting that fine-tuning of the feedback system might be needed.

Integrating burst stimulation protocol into an existing closed-loop control system enabled autonomous path-following navigation of cyborg beetles [37]. This system periodically monitored the beetle’s orientation error relative to a predetermined path (using update intervals of 1.0 and 1.5 s). If the error was significant, a proportional controller (*K*_p_ = 0.5) would adjust the burst stimulation frequency applied to the beetle’s antennae to steer it along the path. Otherwise, a fixed continuous signal stimulates the beetle’s elytra for acceleration. The system’s performance can be illustrated with a typical successful trial (Fig. 3A and Movie S1). Specifically, the proportional controller frequently adjusted the beetle’s orientation by stimulating its 2 antennae, thus reducing the tracking error after each stimulus and highlighting the control efficiency (Fig. 3B). The employed stimulation frequencies spanned from 10 to 40 Hz and were bidirectionally applied to both antennae. Also, relative to its initial orientation, the beetle’s heading angle changed correspondingly with each stimulus, decreasing/increasing (left/right turn) with the stimulation of right/left antenna, consistent with the increase in absolute angular velocity, suggesting efficient stimulation. Despite its simplicity, the system achieved a reliable path completion, with a success rate up to 73% (update interval = 1.0 s; Fig. 4A) and accurate navigation, exhibiting a low tracking error of approximately 12 mm (48% body lengths, update interval = 1.0 s; Fig. 4B). This demonstrated the feasibility of using burst stimulation in feedback control of terrestrial cyborg insects. In optimal trials, the system could promptly complete the predetermined path (a 1,125-mm-long sine curve) in an average of 61 s using only 42 stimuli (update interval = 1.0 s; Fig. 4B).

While the system demonstrates overall high reliability and accuracy, a closer examination of successful trials, i.e., those with path completion, revealed more nuances in the system’s performance and suggested potential improvements. Using a benchmark of 90 s for completing the predetermined sine curve, inspired by similar navigation systems [37], frequent control (update interval = 1.0 s) increased the likelihood of achieving this threshold by around 60%. Particularly, the ratio of “fast” to “slow” successful trials (using the 90-s benchmark) increased from 1.3 to 4.6 when the update interval decreased from 1.5 to 1.0 s, which is likely because frequent control allows the beetle to correct its orientation more readily and be promptly accelerated toward the destination. Furthermore, significant differences existed between the “fast” and “slow” successful trials within each update interval, which favored the former in further developments. In particular, the average path completion time approximately doubled (*t* test, *N* = 5 beetles, *n* = 56 trials, *P* < 0.05; Fig. 4B) in “slow” trials compared to its counterpart. The required stimulation effort also increased by nearly 100% in these trials that failed to pass the benchmark (*t* test, *N* = 5 beetles, *n* = 56 trials, *P* < 0.05; Fig. 4B). Regarding navigation accuracy, there was no significant difference between the 2 groups under frequent control, i.e., around 12 to 15 mm or nearly haft of the body length at both update intervals of 1.0 and 1.5 s (*t* test, *N* = 5 beetles, *n* = 56 trials, *P* > 0.80; Fig. 4B).

**Fig. 5.**
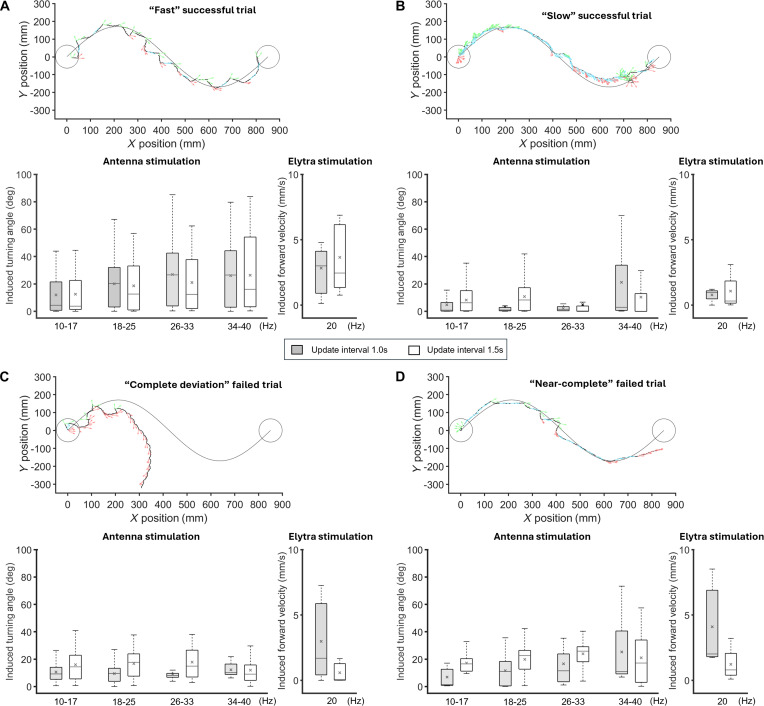
Examination of the beetle’s induced motions in successful and failed navigations. Locomotory response of cyborg beetles among “fast” successful trials (A), “slow” successful trials (B), “complete deviation” failed trials (C), and “near-complete” failed trials (D). In each sub-figure, the top panel displays a representative trajectory of the beetle, the bottom left panel shows the induced turning angle under antenna stimulation at different groups of stimulation frequency, and the bottom right panel displays the induced linear speed under the elytra stimulation. The locomotory response is further divided following the update intervals of 1.0 and 1.5 s. In “fast” successful navigation, the beetle maintains its graded response in the antenna stimulation and a fast linear speed under elytra stimulation. The graded response was significantly diminished in the “slow” successful navigations and “complete deviation” failed navigations, suggesting that monitoring the beetle’s response during navigations is essential.

The beetle’s response to electrical stimulation may explain the differences between the 2 groups of successful trials. The beetle’s graded response was diminished in the “slow” group but preserved in the “fast” group. For example, with an update interval = 1.5 s, the induced turning angle increased from 12.6° to 26.1° as the stimulation frequency increases from 10 to 40 Hz in the “fast” group (Spearman’s correlation, *N* = 5 beetles, *n* > 200 stimuli, *P* < 0.001, *ρ* = 0.34; Fig. 5A). However, in the “slow” group under the same conditions, the turning angle remained relatively flat, increasing only by 19% (Spearman's correlation, *N* = 5 beetles, *n* > 150 stimuli, *P* = 0.31, *ρ* = 0.11; Fig. 5B). This diminished graded response likely reduced the control system’s ability to effectively correct the beetle’s orientation by adjusting the control signal, thus increasing the stimulation number and extending navigation time (Fig. S4 and Movie S2). Furthermore, the beetle’s linear velocity induced by its elytra stimulation is around 73.3% times slower in the “slow” group compared to the “fast” group, further reducing the control system’s efficiency (*t* test, *N* = 5 beetles, *n* > 250 stimuli, *P* < 0.05; Fig. 5A and B). This reduced linear velocity was consistent with the large number of consecutive elytra stimulations used by the system to accelerate the beetle in the “slow” group, which was around 8 stimuli, i.e., 2 times higher than in the “fast” group and even the group of failed trials (Fig. 4C). In other words, the “slow” group required more control effort for less effective results.

Although the control system could overcome these poor locomotory responses and complete its path-following control, one clear objective was to improve these “slow” successful trials, thus ensuring that navigation outcomes were reliable with high success rate, high precision, low tracking, minimal navigation time, and minimal stimulation effort. As demonstrated, a short update interval could help increase the portion of trials within the 90-s threshold. Further improvement would require more efficient maintenance in the graded response and induced linear velocity observed during the antennae and elytra stimulations, respectively. While the relationship between these issues and the increase in stimulation effort was not fully understood, several practical approaches can be explored. Segmenting the beetle’s navigation into fixed-duration slots (e.g., 90 s) with intervening rest periods may help the beetle recover its response to electrical stimulation [38]. In addition to orientation error, monitoring stimulation-related variables [38], such as induced angular velocity, turning angle, and linear velocity, could allow the system to assess the stimulation efficiency and make corresponding adjustments. Besides, tuning the *K*_p_ value of the current control system would be beneficial, as the stimulation frequencies were currently skewed toward the upper range of 40 Hz (third box plot of Fig. 4C), suggesting a suboptimal configuration for the linear proportional controller. Furthermore, given the nonlinear nature of the beetle’s response to electrical stimulation, exploring nonlinear controllers, such as fuzzy logic [60], behavior-based control [38], or even artificial intelligence (AI)-based control [61], may further improve the performance. Implementing burst stimulation and navigation control in more complex environment, such as sandy terrain, an effective platform to study the on-field locomotion and adaptability of terrestrial insects [42], would also be considered in future work to verify their practical efficiency. Investigating long-term effectiveness of burst stimulation would be beneficial for real-life applications.

Variations in locomotion performance and turning responses under burst stimulation were comprehensively analyzed across 5 individual cyborg beetles (Fig. S5). Using a similar categorization of successful and failed trials as presented in Fig. 4A, each beetle demonstrated a success rate ranging from approximately 62% to 94% (*N* = 5 beetles, *n* = 60 trials; Fig. S5A), i.e., including both “fast” and “slow” trials. This wide variation in locomotion performance was notably influenced by the occurrence of “near-complete” trials, where beetles with lower overall success rates exhibited higher proportions of this failure case. For example, beetles 1 and 5 achieved success rates of ~62% in autonomous navigation while having ~38% and ~24% of “near-complete” trials, respectively (Fig. S5A). In addition, “complete deviation” trials could be considered as a generalized failure trend among cyborg insects, with 4 of 5 beetles experiencing this type of failure at a rate of approximately 6% to 10%. Evaluating metrics similar to those in Fig. 4B revealed that the efficiency of the closed-loop control system under burst stimulation also varied among 5 beetles. Considering the navigation time per trial, beetles 1 to 3 and 5 demonstrated more effective performance under burst stimulation, with average navigation times remaining below ~100 s [analysis of variance (ANOVA), *N* = 5 beetles, *n* = 47 trials, *P* < 0.025; Fig. S5A]. Moreover, over 42% of their trials were successfully completed within 90 s (Fig. S5A). Meanwhile, beetle 4 required up to ~149-s average time to reach the final destination (Fig. S5A). Such navigation times align with the number of stimuli required per trial. Beetles 1 to 3 and 5 reached the destination with fewer than ~50 electrical stimulations. In contrast, beetle 4 required significantly more stimuli, with an average value of ~77 (ANOVA, *N* = 5 beetles, *n* = 47 trials, *P* < 0.05; Fig. S5A). This aberrant behavior may be attributed to a lower slope of graded turning response within the 10- to 40-Hz frequency range of beetle 4 (Fig. S5B). On the other hand, a consistent graded response was illustrated across 4 remaining beetles, i.e., the slopes of linear regression lines have the maximum deviation of ~25% for induced turning angle and ~39% for induced angular velocity (linear regression, Spearman’s correlation, *N* = 5 beetles, *n* = 47 trials, *P* < 0.05, *ρ* > 0.32; Fig. S5B). In terms of accuracy in autonomously tracking a predefined path, beetles 2 to 5 exhibited consistent average tracking errors of ~9 to 13 mm, whereas beetle 1 showed a significantly higher error of ~22.5 mm (ANOVA, *N* = 5 beetles, *n* = 47 trials, *P* < 0.001; Fig. S5B). The high tracking error may be due to the strong graded response exhibited by beetle 1, which could potentially lead to overshoots during tracking.

In summary, although an exact threshold for habituation cannot be defined under all conditions due to variability in factors such as stimulation intervals, free-walking periods, and individual recovery times, which remains a major challenge for biohybrid platforms, several criteria can be proposed to assess controllability before habituation occurs. Specifically, for successful tracking of a predefined path, one may consider the navigation effort (number of stimulations per trial) and the frequency distribution of both “fast” and “slow” trials (Fig. 4), as well as the deterioration rate of turning-angle responses across frequency ranges (Fig. 2). To successfully guide cyborg beetles along a predefined path, the total number of stimulations should remain below 50, with high-frequency pulses at 40 Hz accounting for no more than 50% of a trial (Fig. 4), i.e., the threshold is defined based on the control effort and frequency distribution of the “fast” successful trials. Although up to 120 stimulations can still yield successful navigation, this increases habituation, prolongs navigation time, and accelerates response deterioration in subsequent trials. Considering the deterioration rate of turning angle (Fig. 2), responses typically fall below 5° after 7 to 10 stimulations at 10 to 30 Hz, which can serve as the unilateral stimulation threshold before habituation in this frequency range. At 40 Hz, “complete deviation” trials occurred after only 4 to 5 stimulations, suggesting this as the upper limit for consecutive high-frequency inputs. Recognizing that the turning response can deteriorate after repeated unilateral stimulation of a single antenna, 2 potentially effective strategies can be considered: (i) enhancing the control logic of the autonomous navigation system and (ii) adaptively varying burst parameters to mitigate directional drift. For the first strategy, additional control modules could be integrated into the existing framework. For example, low-frequency stimulation could be applied to the contralateral antenna to elicit a corrective U-turn when no significant turning response is detected from the stimulated antenna. Alternatively, brief simultaneous stimuli of both antennae could be used to temporarily immobilize the insect, allowing it to stabilize before resuming navigation. For the second strategy, adaptive modulation of burst parameters, such as varying stimulation frequency or interburst intervals, may reduce the effects of consecutive high-frequency stimulation, thereby promoting recovery and maintaining more consistent turning behavior.

### Insights into navigation failures of cyborg beetles

Analyzing navigation failures could provide valuable insights for improving the reliability of autonomous navigation in cyborg beetles. The navigation failures could be classified into 2 groups, “near-complete” and “complete deviation”. “Near-complete” failures exhibit trajectories that mostly follow the predetermined sine curve but fail to reach the destination before the experiment terminates (Fig. 4A, Fig. S7, and Movie S4). In contrast, “complete deviation” failed trials showed significant deviations from the predetermined path, either from the start or after a period of tracking (Fig. 4A, Fig. S6, and Movie S3). Analysis of the stimulation signals revealed significant differences in unilateral stimulation applied to the beetle’s antenna, meaning consecutive stimulation of one antenna to steer the beetle along the path (Fig. 4C). The “near-complete” failures exhibited an average of 7 consecutive unilateral stimuli per trial. In contrast, the group of “complete deviation” failures showed a significantly higher average of 13 consecutive unilateral stimuli, ranging from 11 to 15.

This bias in stimulation might contribute to navigation failures, especially given that successful trials involved significantly less consecutive unilateral stimulation, i.e., around 4 consecutive unilateral stimuli per trial (Fig. 4C). Furthermore, in the “complete deviation” failures, which were more severe than “near-complete” failures, the consecutive unilateral stimulation often occurred at high frequencies, skewing the overall distribution of stimulation frequencies to the right (Fig. 5C and Fig. S6). This was likely because, as the beetle deviated from the path, its orientation error increased, leading to higher stimulation frequencies generated from the proportional controller to reorient it. In these trials, the beetle appeared to favor movement in the same direction as the stimulated antenna. The beetle persistently moved rightward after briefly being steered leftward by consecutive stimulation of its right antenna. Such behavior may resemble the wall-following behavior observed in terrestrial insects, where tactile/mechanical stimulation of one antenna guides their movement along walls [11,34]. Insects can also exhibit preferred movement directions influenced by individual preferences or environmental factors [53,62]. In these failure cases, such behaviors appeared to conflict with the control system’s objective, negatively impacting the outcome. However, burst stimulation proved to be a more effective protocol than the conventional continuous waveform, as “complete deviation” cases occurred more frequently and at earlier stages of the trial under continuous stimulation (Fig. S8).

The beetle’s response to electrical stimulation might also contribute to navigation failures. Like the “slow” successful trials group, the graded response to the antenna stimulation was distorted in the failed trials (Fig. 5C). In the “complete deviation” failures, increasing the stimulation frequency from 10 to 40 Hz did not increase the induced turning angle, which remained relatively flat at approximately 15 to 18° (update interval = 1.0 s). This distortion might be due to unilateral bias in the antenna stimulation at high frequencies. The distortion in the graded response appeared less severe in the “near-complete” failures, which might explain their near-successful path-tracking attempts. Increasing the stimulation frequency from 10 to 33 Hz increased the induced turning angle by an average of 12° (update interval = 1.0 s; Fig. 5D). Given that these trials successfully track most of the predetermined sine path, increasing the navigation time might enable the beetle to reach the destination (Fig. S7).

As discussed, the diminishing graded response requires further investigation into its causes, and it can potentially be addressed through practical approaches such as frequent control, response monitoring, control parameter tuning, implementing nonlinear controls, and/or incorporating navigation with rest periods. As increasing the navigation time may improve the likelihood of success in “near-complete” failures, implementing the proposed approaches could further enhance the timeliness and efficiency of navigation outcomes. For the “complete deviation”, further research into the cause of the beetle’s persistent movement direction might be essential. This issue could be tackled by improving the control system, e.g., integrating additional control strategies to address the nonresponse behavior observed during unilateral antennal stimulation. For instance, some natural behaviors, such as obstacle negotiation abilities [11,12], can be exploited to enhance navigation, whereas others that may negatively impact the navigation result, such as the tendency to stop in corners or near impassable obstacles, can be detected using additional sensors and avoided with adaptive adjustment of control strategies [13,20,39,41]. Herein, a similar approach could be applied to foresee the beetle’s persistent movement direction to manage their impact on navigation reliability. For example, an IMU could be used to monitor the beetle’s orientation tendencies during stimulation-free periods to classify its preferred direction. A more straightforward approach is to track the number of consecutive unilateral stimulations applied to the beetle antenna as an indication of the potential appearance of its persistent movement direction. A threshold of 4 observed in successful trials can be used to detect such biased motion. Upon detection, the navigation system could update the beetle’s path to accommodate its preferred movement or attempt to steer the beetle using the other antennae can be used to detect such biased motion. Then, the navigation system could update the beetle’s path to accommodate its preferred movement or attempt to steer the beetle using the other antenna. Furthermore, modulating burst, such as adjusting the frequency or interburst interval, may help the insect recover from the effects of consecutive high-frequency stimuli and maintain consistent turning behavior.

### Burst stimulation across insect species

Despite the diversity in shape and size, terrestrial insects exhibit similar turning behavior when using their antennae as sensory guides to detect obstacles, gradients, and directional cues in their environment. For example, cockroaches use their long, mobile antennae to sense surfaces and initiate rapid turns to avoid collisions or navigate around barriers [12,36,63], often adjusting their orientation based on antennal contact duration and angle. Ants rely on their antennae not only to follow pheromone trails but also to detect trail bifurcations, adjusting their turning behavior accordingly to maintain route fidelity [64]. Similarly, stick insects use antennal contact to explore terrain and initiate precise turning movements when encountering uneven surfaces or obstacles [65]. These examples highlight the role of antennae as active sensors that enable various insects to perform fine-tuned turning maneuvers essential for terrestrial navigation. As a result, electrical stimulation of insects’ antennae has been demonstrated as an effective method to control their locomotion in various insect species, including darkling beetle (*Z. morio*) [50], Madagascar hissing cockroach (*G. portentosa*) [38], American (*Periplaneta americana*) and discoid (*Blaberus discoidalis*) cockroaches [29], and migratory locust (*L. migratoria manilensis*) [28]. Furthermore, using burst stimulation for turning control more closely mimics the natural burst-like neural activity observed in the antennae of intact terrestrial insects [35] when typical extracellular recordings show that stimulation of the intact antenna elicits burst activity in descending mechanosensory interneurons [33]. Employing burst stimulation at sensory organs including antennas to control navigation enhances the control outcome with higher success rate and more naturistic responses in various animals [50–52]. Therefore, the burst stimulation protocol proposed in this paper could potentially be implemented on other insect platforms to enhance the controllability and sustainability of terrestrial cyborg insects.

## Conclusion

This study confirms the role of burst stimulation in sustaining the locomotory response of terrestrial cyborg insects to electrical stimulation, thereby enhancing the reliability of the control system. The burst stimulation protocol increases the induced motion while slowing down habituation. Combining this with existing methods, such as monitoring implant quality [46] or controlling stimulation amplitude [38], we would be able to form a multi-layered protection strategy for the sustainability of locomotion control in terrestrial cyborg insects, facilitating real-world deployment. Burst stimulation can be integrated effectively into feedback control systems for autonomous navigation of terrestrial cyborg insects due to the graded nature of the induced motions. While a simple proportional controller can achieve a highly successful path-following with high tracking accuracy, there is still room for improvement. For example, stimulation quality (e.g., graded response), quantity (e.g., the number of consecutive unilateral stimuli), and the beetle’s navigation behaviors (e.g., wall-following) can be monitored and used as feedback for the control system. Further investigation into the characteristics of burst stimulation, such as burst number and rest interval, may also yield additional benefits for enhancing locomotory responses and navigation performance.

## Data Availability

All data needed to evaluate the conclusions in the paper are present in the paper or in the Supplementary Materials.
