# Supplementary material for: Burst Stimulation for Sustained Locomotion Control and Autonomous Navigation of Terrestrial Cyborg Beetles
Source: Cyborg Bionic Syst. 2026 Mar 9;7:0537. doi: 10.34133/cbsystems.0537 (PMC12968394; doi:10.34133/cbsystems.0537)
Supplement: Supplementary 1 — Figs. S1 to S8 Movies S1 to S4 [file cbsystems.0537.f1.zip › Supplementary_Materials.docx]

SUPPLEMENTARY MATERIALS

## **Supplementary Figures**


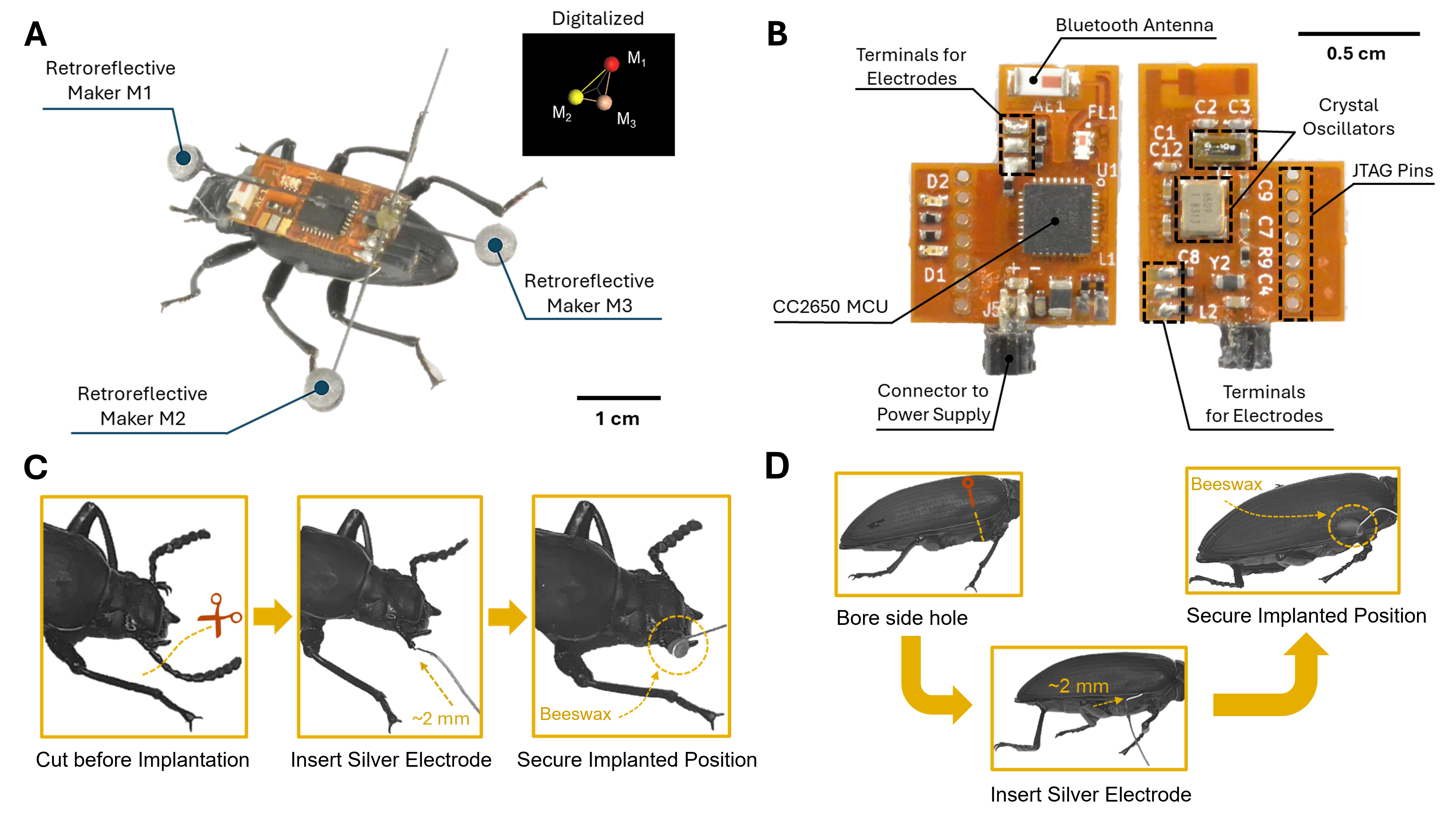


**Figure S1.** **Cyborg beetle with wireless backpack and markers for navigation experiment.** **(A)** Three retroreflective markers (~5 mg, radius 3 mm) were attached to the wireless backpack of every experimental cyborg beetle. These markers form an object (M1, M2, M3) in the motion capture software and provide location data ($x$, $y$) to the control system. **(B)** Top (left) and bottom (right) view of the wireless backpack. The backpack relies on the system on chip (SoC) microcontroller (MCU) CC2650 (Texas Instruments) with Bluetooth communication. Eight terminals can be used for electrical stimulations while the power supply port can connect to a micro battery or a benchtop power supply through loose tethered wires. The JTAG pins are used for uploading the firmware to the MCU and cut off when needed. **(C)** Antenna implantation for turning control. Silver electrodes (70 µm diameter, bare; 100 µm diameter, coated; A-M Systems) were inserted ~2 mm in depth after cutting the antennas to reveal the implanted positions **(D)** Elytra implantation for accelerating control. Two silver electrodes was inserted into elytra along the thorax sides, the implanted positions were created using insect pin.

**Figure S2. The response of terrestrial cyborg beetles to burst stimulation.** The induced turning angle **(A)**, induced angular velocity **(B)**, and induced forward velocity **(C)** of the beetle when the left (green) and right (red) antenna were stimulated using burst protocol. The lines represent means across trials while shaded regions indicate standard error. Green lines and regions indicate left antenna stimulation while red ones indicate right antenna stimulation. Black lines and grey regions indicate period without stimulation.

**Figure S3. The responses of terrestrial cyborg beetles to continuous stimulation**. The induced turning angle **(A)**, induced angular velocity **(B)**, and induced forward velocity **(C)** of the beetles when the left (green) and right (red) antenna were stimulated using continuous pulse train. The lines represent means across trials while shaded regions indicate standard error. Green lines and regions indicate left antenna stimulation while red ones indicate right antenna stimulation. Black lines and grey regions indicate period without stimulation.

**Figure S4.** **Performance of a representative “slow” successful navigation. (A)** Representative trajectory of a cyborg beetle autonomously navigating along the path in more than 90 s. Arrows indicate heading of the beetle with right (red)/ left (green) antenna or elytra (blue) stimulation. **(B)** The performance of the cyborg beetle when navigating along the path. The top row shows zoom-in windows of the beetle response in **(A)**. The second row (dark yellow line) shows the instantaneous distance between the beetle and the center of destination region. The third row (purple line) presents stimulation frequencies and stimulation sites. The fourth row (dark red line) shows the instantaneous tracking error between the beetle trajectory and experimental route. The fifth row (blue line) presents the instantaneous heading angle. Fifth and sixth rows (black and grey lines) are instantaneous angular velocity, and forward velocity, respectively. A low-pass filter (normalized passband frequency = 0.01) was applied for the angular velocity. There are significantly high number of stimuli along the trajectory (*n* = 120 stimuli) comparing to the representative “fast” successful case (*n* = 30 stimuli). This high number of stimuli could be due to habituation that led to response degradation (Fig. 4C) and prolonged the navigation time to 204 s. However, the control system was able to navigate the cyborg beetle to the destination regardless habituation.


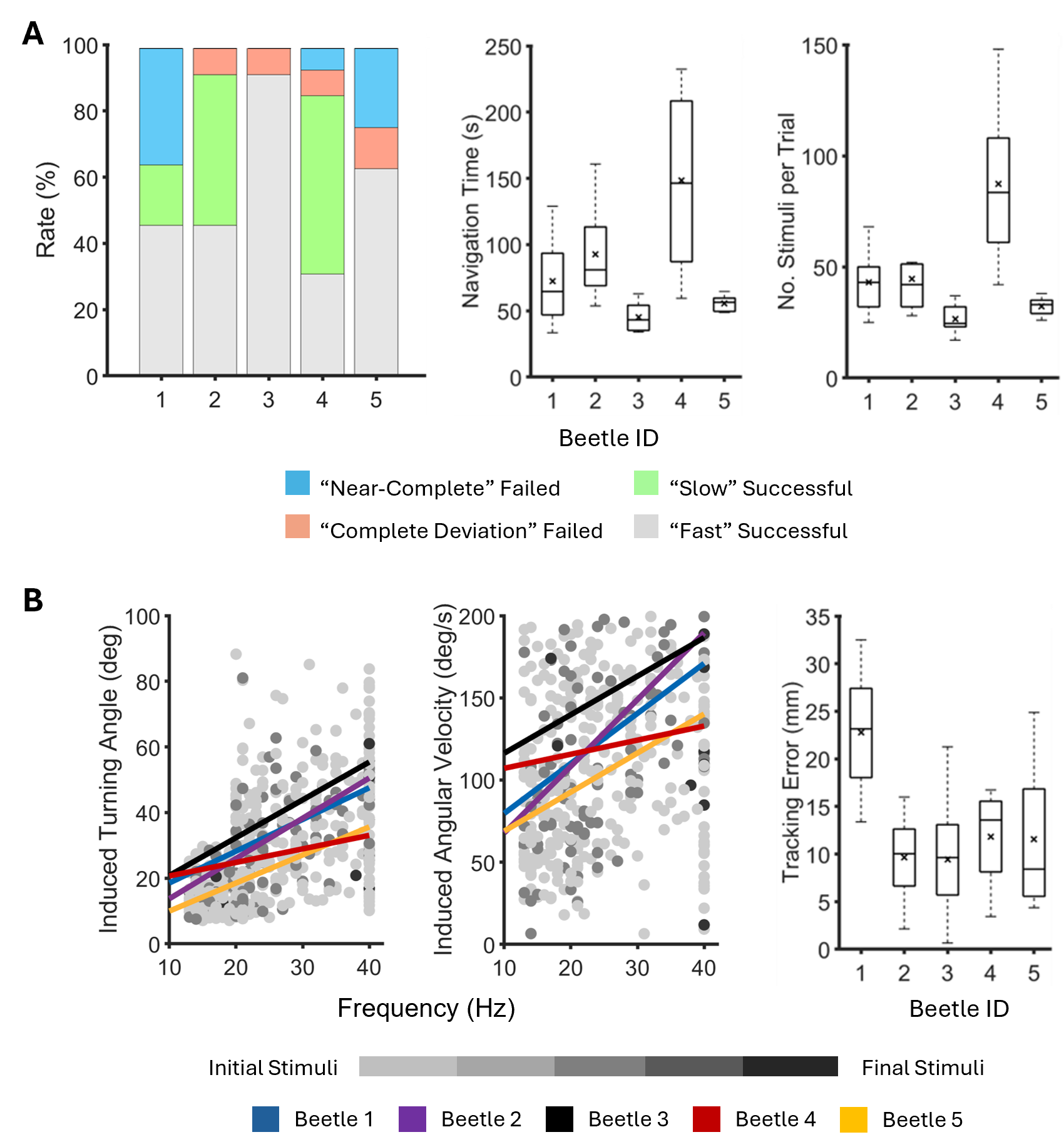


**Figure S5. Variation in navigation performance among individual beetles. (A)** Efficiency of the feedback control system in individual cyborg beetles (*N =* 5 beetles, *n =* 60 trials). The ratio of successful and failed navigations (left) shows clear difference across individual beetles. Trials are classified into four groups: “fast” successful trials (grey), in which the beetle completes the trial within 90 seconds; “slow” successful trials (green), completed in more than 90 seconds; “near-complete” failed trials (blue), where the beetle partially follows the path but does not reach the final destination; and “complete deviation” failed trials (red), where the beetle significantly deviates from the intended path. Navigation time (middle) and the number of stimuli per trial (right) for each beetle are presented as boxplots, with cross marks indicating the means and horizontal lines representing the medians. **(B)** Turning responses (left) (*N =* 5 beetles, *n =* 1272 stimulations) and tracking error (right) of individual beetles. Graded turning responses, specifically induced turning angles and angular speeds, are depicted using coloured lines, which represent linear regressions of the induced response as a function of applied stimulation frequency. Actual tracking errors (right) are illustrated using boxplots, consistent with the representation of navigation time.

ssss

**Figure S6.** **Performance of a representative “complete deviation” failed navigation. (A)** Representative trajectory of a cyborg beetle autonomously navigating along the path with 11 unilateral consecutive stimulation applied to its left antenna. Arrows indicate heading of the beetle with right (red)/ left (green) antenna or elytra (blue) stimulation. **(B)** The performance of the cyborg beetle when navigating along the path. The top row shows zoom-in windows of the beetle response in **(A)**. The second row (dark yellow line) shows the instantaneous distance between the beetle and the center of destination region. The third row (purple line) presents stimulation frequencies and stimulation sites. The fourth row (dark red line) shows the instantaneous tracking error between the beetle trajectory and experimental route. The fifth row (blue line) presents the instantaneous heading angle. Fifth and sixth rows (black and grey lines) are instantaneous angular velocity, and forward velocity, respectively. A low-pass filter (normalized passband frequency = 0.01) was applied for the angular velocity. Although the beetle shows clear response to the burst stimulation, the value of absolute heading angle from the last twenty seconds indicates much lower turning response comparing to earlier stimuli, which could be due to habituation through consecutive and high frequency stimuli. Despite clear responses to electrical stimulation in the last 20 s, the beetle’s forward velocity reduces significantly, possibly due to its natural motion preference, such as wall-following. Such behaviour suggests that it is crucial to reduce the number of consecutive stimuli when navigating cyborg beetles.

**Figure S7.** **Performance of a representative “near-complete” failed navigation. (A)** Representative trajectory of a cyborg beetle autonomously navigating along the path but could not reach the destination before the experiment was terminated. Arrows indicate heading of the beetle with right (red)/ left (green) antenna or elytra (blue) stimulation. **(B)** The performance of the cyborg beetle when navigating along the path. The top row shows zoom-in windows of the beetle response in **(A)**. The second row (dark yellow line) shows the instantaneous distance between the beetle and the center of destination region. The third row (purple line) presents stimulation frequencies and stimulation sites. The fourth row (dark red line) shows the instantaneous tracking error between the beetle trajectory and experimental route. The fifth row (blue line) presents the instantaneous heading angle. Fifth and sixth rows (black and grey lines) are instantaneous angular velocity, and forward velocity, respectively. A low-pass filter (normalized passband frequency = 0.01) was applied for the angular velocity. The responses of the cyborg beetle to right antenna stimulation are lower than that of left antenna stimulation, leading to higher number of consecutive stimuli compared to that of “fast” successful navigation (Fig. 4C). These responses indicate habituation to right antenna stimulation and a strategy to detect this with increasing left antenna stimulation could enhance navigation efficiency.


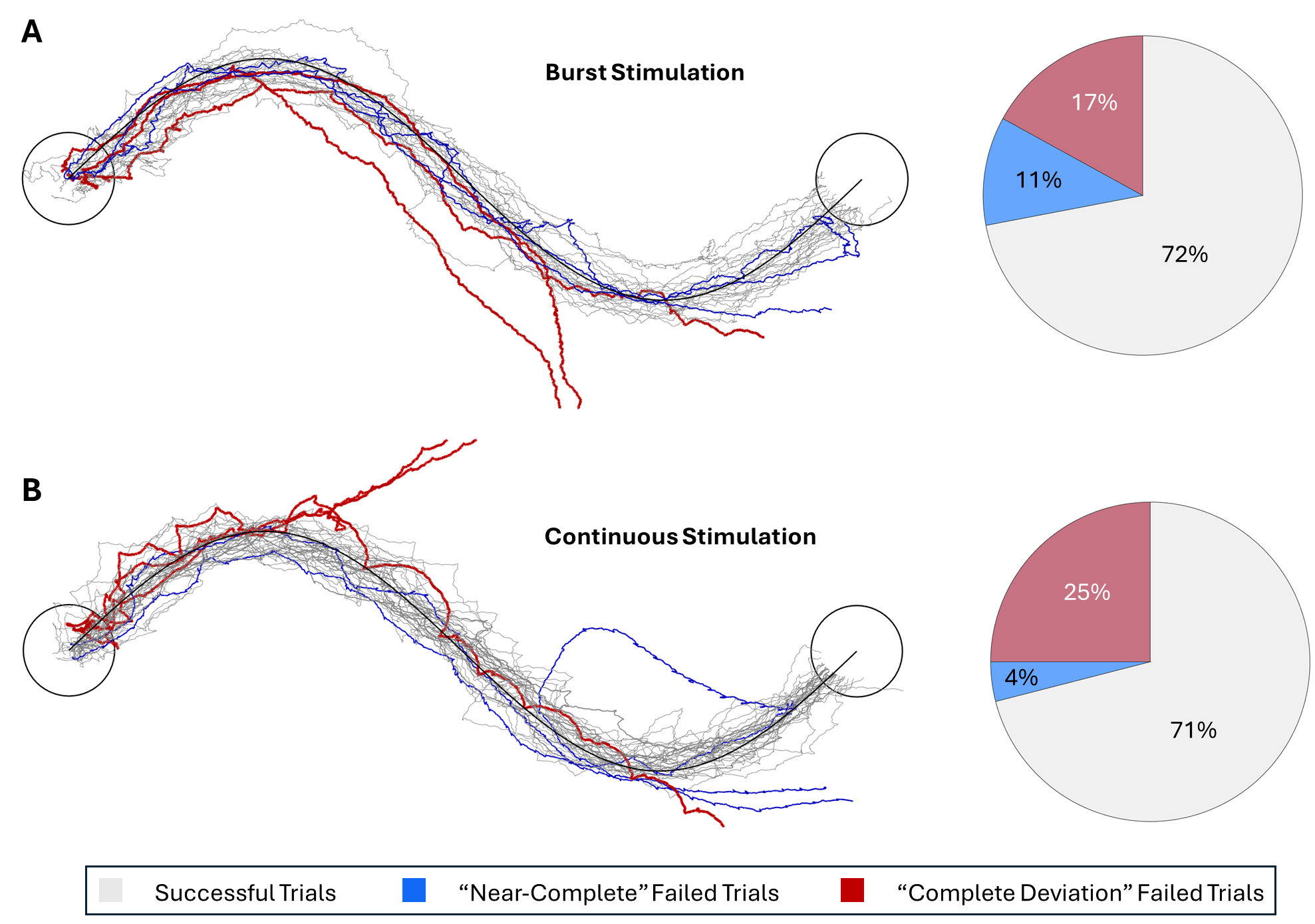


**Figure S8. Effectiveness of both stimulation protocols when implemented in feedback control system.** Trajectories and success rates of experimental trials under burst stimulation (*N* = 5 beetles, *n* = 56 trials) (A) and continuous stimulation (*N* = 20 beetles, *n* = 35 trials) (B). “Complete-deviation” failures typically occurred earlier in the trial than under continuous stimulation. Although burst stimulation did not increase the overall success rate, the number of “complete-deviation” cases decreased when this protocol was employed. Moreover, because burst stimulation produced a higher proportion of “near-complete” trials, it shows greater potential for improved performance: if these near-complete trials were brought to completion, the success rate would rise to approximately 83%, exceeding that of conventional continuous stimulation.

## **Supplementary Movies**

**Movie S1.** Representative demonstration of “fast” successful navigation. The beetle completes tracking the path and reaches the destination within 90 s.

**Movie S2.** Representative demonstration of “slow” successful navigation. The beetle completes tracking the path and reaches the destination after 90s.

**Movie S3.** Representative demonstration of “complete deviation” failed navigation. The beetle significantly deviates from the path. More than 11 unilateral consecutive stimulations applied to its right antenna.

**Movie S4.** Representative demonstration of “near-complete” failed navigation. The beetle partially tracks the predetermined path, but it can’t reach the destination before the experiment is terminated.
